# Strategic Guidance and Technological Solutions for Human Resources Management to Sustain an Aging Workforce: Review of International Standards, Research, and Use Cases

**DOI:** 10.2196/27250

**Published:** 2022-07-21

**Authors:** Ann Kathrin Wissemann, Sabrina Winona Pit, Patrick Serafin, Hansjürgen Gebhardt

**Affiliations:** 1 Institute of Occupational Health, Safety and Ergonomics (ASER) Eingetragener Verein Wuppertal Germany; 2 Work Wiser International Lennox Head Australia; 3 University Centre for Rural Health University of Sydney Lismore Australia; 4 School of Medicine Western Sydney University Lismore Australia

**Keywords:** workforce, HR, aging, older people, standards, international standards, occupational health, blockchain, AI, ICT, strategy

## Abstract

**Background:**

New technologies offer opportunities to create a healthy, productive, and capable aging workforce. There is little research from an organizational perspective about how technology can help create a sustainable aging workforce.

**Objective:**

This study aims to (1) explore how technological solutions in organizations can help create and maintain a healthy, productive, and capable aging workforce; and (2) provide recommendations and strategic guidance that benefit both the aging worker and the organization.

**Methods:**

International standardization practices, ethical frameworks, collaborative research, and use cases are used to demonstrate how technological solutions can be translated into practice and formed the basis for the development of a set of recommendations to create and maintain a sustainable aging workforce.

**Results:**

Organizations need to look at aging through different lenses to optimize an age-inclusive workforce rather than viewing it by chronological age alone. International standards in technology, human resources management, and aging societies can form part of the solution to improve aging workforces. Digitalization of workplaces, digital literacy, innovation, intergenerational collaboration, and knowledge management form important elements of the international standard on age-inclusive workforce. Using internationally agreed ethical frameworks that consider age bias when designing artificial intelligence–related products and services can help organizations in their approach. Age bias in artificial intelligence development in the workplace can be avoided through inclusive practices. No blockchain application was found yet to improve the aging workforce. Barriers to blockchain adoption include fear of layoffs, worker resistance and lack of blockchain competence, worldwide adoption, support, and funding. Integrating blockchain into the internet of things may allow for improved efficiencies, reduce cost, and resolve workforce capacity problems. Organizations could benefit from implementing or funding wearable technologies for their workers. Recent tools such as the Ageing@Work toolkit consisting of virtual user models and virtual workplace models allow for the adaptation of the work processes and the ergonomics of workplaces to the evolving needs of aging workers. Lastly, selected use cases that may contribute to sustaining an aging workforce are explored (eg, the Exposure-Documentation-System, wireless biomedical sensors, and digital voice notes).

**Conclusions:**

The synergy of international standardization and ethical framework tools with research can advance information and communication technology solutions in improving aging workforces. There appears to be a momentum that technological solutions to achieve an age-inclusive workforce will undoubtedly find a stronger place within the global context and is most likely to have increased acceptance of technological applications among aging workers as well as organizations and governments. International standardization, cross-country research, and learning from use cases play an important role to ensure practical, efficient, and ethical implementation of technological solutions to contribute to a sustainable aging workforce.

## Introduction

### Aging and Working Population Changes

In many developed and developing countries, the aging population is increasing due to advances in medicine and public health as well as socioeconomic developments. The number of people aged 65 years or over in the global population will double from 703 million in 2019 to 1.5 billion in 2050 [1].

This demographic change represents a challenge for the working environment as the workforce is aging [2]. In 2018, 20% of the working population in the European Union was aged 55 and over, with one-third of them being over 60 years [3]. In the United States, it is estimated that by 2024, 25% of the workers will be 55 years and older. One-third of them will be over 65 years [4]. The old-age dependency ratio (OADR) is also expected to rise sharply. The OADR describes the ratio of people aged 65 and over (old ages) to people aged 20-64 years (working ages). In Europe and North America, the OADR is projected to rise from 30 in 2019 to 49 in 2050 per 100 working age persons. By 2050, many Asian and Latin American countries are projected to join European countries in having high OADRs [1]. For example, Japan is expected to have the highest OADR in the world of 81, followed by the Republic of Korea at 79, and Spain at 78, while China and Taiwan will have an OADR of 71 per 100 working age persons.

### Aging Workforce and Technological Changes

Simultaneously with demographic changes, the fourth industrial revolution (Industry 4.0) is rapidly developing. Organizations aim to exploit developments in the field of artificial intelligence (AI), the internet of things (IoT), and collaborative robots. With changing market demands, and the shift from mass production to increasingly specialized and customized products, the complexity in production and work tasks will increase. Work will increasingly take place in automated factories with cyber-physical production systems, where machines, products, and workers will be interconnected [5]. Work tasks and processes will become increasingly complex such as supervision of machines, and will increasingly require problem-solving skills. Good knowledge management and information and communication technology (ICT) become increasingly important as new work tasks require a higher level of knowledge and information processing ability.

While some jobs may disappear due to technology, new ones will emerge [6]. Furthermore, new organizational business models, such as platform work (eg, Uber), and evolving worker preferences, such as remote work [6], have led to technological changes in the workplace. COVID-19 has also demonstrated the “forced innovation” such as the implementation of telehealth [7], increased use of videoconferencing, and technological solutions created during COVID-19 to support an aging workforce [8].

### Benefits and Downsides of Technological Advances and an Aging Workforce

An age-inclusive workforce can increase the competitive advantage through increased engagements and performance [9]. Conversely, there is a stream of thought that age diversity is harmful for organizational productivity, which was confirmed in a Belgian sample of 2431 organizations in the private sector [10]. However, van Ours and Stoeldraijer [11] did not find any relationship between increasing age and a pay-productivity gap in the Dutch manufacturing industry. And, more recently, a 2019 Estonian study demonstrated that older workers are as productive as younger workers [12]. Overall, the evidence appears conflicting [13] and a meta-analysis has demonstrated that the relationship between productivity and workers’ age is not yet clearly established empirically [14].

Broadly, quality work means that people can be productive at work, add value to the organization, have a decent income, feel secure in the workplace and socially included, and are able to participate in decisions that affect their working environment.

Provided that work is quality work, additional benefits of prolonging working lives for older people include work being good for health [15], allowing social participation, and providing financial independence. When workers age, some physical and mental capabilities (such as visual or reaction time) decline [16], while professional knowledge and experience increase. ICT can play a role to ensure quality work for aging workers. To date, technology has mostly replaced both physical and cognitive repetitive tasks through computer programming, whereas it has become complementary to the performance of nonroutine cognitive tasks for humans [14].

On the one hand, ICT can improve working conditions, for example, by reducing loads, shortening commuting time, or assisting in activities. ICT can compensate for some of the decreasing capabilities of older workers or reduce risks in terms of hazardous tasks, and protect older workers during pandemics by allowing remote working [7]. Ergonomics plays an important role in creating aging-appropriate work designs to meet the needs of workers. Including aging workers in the design process can contribute to achieving this goal [17]. On the other hand, traditional jobs may be lost, continuous and more rapid upskilling in technology is needed, and workers may have difficulties in the use of new technologies. Furthermore, technology may decrease autonomy at work [6], which is an important determinant for quality work. Currently, the literature is inconclusive about the negative aspects of technology on working conditions [6].

Another consideration is that younger workers’ knowledge about new ways of working and new technologies is often perceived to be more explicit. But the tacit knowledge of older workers gained through experience is equally important, especially for performing complex tasks. Research has shown that older people perform more consistently on cognitive tasks than younger people [18]. Older workers have competencies that younger workers may not have developed yet. Thus, the skills of younger and older workers complement rather than substitute each other [19].

Nagarajan and colleagues [17] conducted a systematic review of 122 studies (1990-2018) to identify organizational factors that contribute to sustaining an aging workforce. Notably, they found that technological tools were the least researched factor that contributes to a sustainable aging workforce. Only 4.9% (6/122) of the research focused on technology compared with the other 4 main factors, including human capital (40/122, 32.7%), institutions (32/122, 26.2%), human resources management (29/122, 23.7%), and health (15/122, 12.3%). The authors suggested future work is needed in the area of technological tools to improve productivity in an aging workforce [17]. Our paper seeks to address this gap by exploring how technology can be used to improve the aging workforce.

### Objectives

This paper aims to (1) explore how ICT solutions in organizations can help create and maintain a healthy, productive, and capable aging workforce; and (2) provide recommendations and strategic guidance that benefit both the aging worker and the organization.

## Methods

Different sources of data are drawn upon that point to the benefits of and how ICT can help create and maintain a healthy, productive, and capable aging workforce that adds value to both the organization and the aging workforce. The focus of attention was on using different lenses on aging, international standardization, ethical frameworks, international collaborative research, and practical use cases. Information was gathered on international efforts in the field of aging workforce based on research in technical literature, professional journals, and internet articles. We searched the International Organization for Standardization (ISO) browsing platform [20] and contacted international experts.

A brief literature review was conducted in Web of Sciences (2010-2021) using the following keywords: information communication technology and (human resources or workforce or employee or worker) and (aging or older or old or aging or mature). Searches were restricted to English publications.

Following this, several distinct areas of aging workforce and ICT were further explored. First the importance of using different lenses toward aging was examined, and a multidimensional perspective to optimize an age-inclusive workforce was presented. Second, international standards directories were examined to determine how international standardization contributes to a sustainable aging workforce. Third, increased digitalization leads to an increased need to understand the ethical requirements and implications of these changes in the workplace. It also requires us to understand how ethics can contribute to a sustainable aging workforce. Therefore, ethical frameworks and standards that deal with ethics in relation to ICT and an aging workforce were examined. Fourth, novel research in the area of aging workforce and ICT solutions were examined to demonstrate how international collaborative research can contribute to a sustainable aging workforce. Lastly, several use cases were studied to show how ICT solutions can be translated into practice and applied.

Based on the steps above, a set of recommendations was developed for organizations to provide strategic guidance and practical solutions to improve the aging workforce.

## Results

### Context of an Aging Workforce and ICT

A Web of Science literature search between 2010 and 2021 identified 324 papers; of these, 27 abstracts were selected for further review and 13 were finally included in this review. Themes that emerged centered around the impact of age and ICT on health-related outcomes (n=6); the impact of technology on the demand for older workers (n=3); the relationship between aging, ICT investment, and productivity (n=1); age and the use of social media for work purposes (n=1); and lastly the impact of ICT on the rise of nontypical employment and associated occupational health and safety (OHS) consequences (n=1).

Six papers focused on the impact of age on ICT and health-related outcomes. Overall, studies found no relationship between age, ICT, and health. Berg-Beckhoff et al [21] conducted a systematic review and concluded that although ICT leads to more burnout, there was no linear relationship between age and technostress and burnout. Arvola and colleagues [22] also found that there is no difference in well-being and mental health between older workers who do tele-work and those who do not. Borle and co-workers [23] also reported that, among German workers, ICT does not negatively affect the health and social life of older workers, but they did find that digital work intensification overall is associated with worsening mental health and work ability but not physical health.

By contrast, Hauk et al [24] reported that increasing age led to reduced technostress, based on longitudinal data among 1216 employees. The authors explain that this relationship is influenced by the level of work engagement, and that because older workers tend to have higher work engagement compared with their younger counterparts, they have less technostress. Setyadi and colleagues [25] went a step further and explored how different concepts of aging, including cognitive age and chronological age, influence the effect of technostress on work satisfaction, performance, and intention of long-term ICT use. They concluded that the higher the cognitive age (coined as “young spirited workers”), the less technostress the worker experienced. This highlights the need for organizations to look at different dimensions of aging. Furthermore, technostress was mainly influenced by techno overload, uncertainty, and insecurity as well as workers’ intention to continue to use technology. Carlotto et al [26] found among Brazilian ICT workers that professionals aged between 35 and 60 years reported a greater identity with their career, higher satisfaction with life, and less technostress. In summary, it appears that age influences one’s career identity and work engagement in a positive manner, and therefore can possibly lead to reduced technostress.

Three papers examined the impact of technology on the demand for older workers, with 2 focusing on high-income countries [27,28] and one on a lower-income country (Pakistan) [29]. Peng et al [27] found that in 9 European countries between 1970 and 2007, ICT led to a decrease in the demand for older workers. The authors reported that some deskilling of older workers took place, which influences the demand of these older workers and suggests that these trends can be alleviated somewhat through activities such as wage settings and collective bargaining agreements [27]. This may require government-level support. Similarly, Blanas and co-workers found, in 10 high-income countries and 30 different industry sectors, that software and robots reduced the demand for young, lower-skilled, and female workers, notably in the manufacturing industry [28], whereas it increased the demand for older, high-skilled, and male workers, particularly in the service industry. Both studies confirm that lower-skilled and routine human tasks are replaced by ICT. Lastly, Hanif et al [29] looked at 295 older workers in the ICT sector in Pakistan and found that age, gender, and lower health status are barriers to sustainable employability, while technical qualifications facilitate sustainable employability.

A 2020 Japanese and Korean study explored the complex relationship between aging, ICT investment, and productivity, and found that in Japan and Korea aging has a positive effect on labor productivity in organizations with a high level of ICT capital investments [30]. However, compared with younger workers, in Japan a higher proportion of lower-educated older workers have a positive impact on productivity, whereas in South Korea a larger proportion of higher-educated older workers have a positive impact on productivity. The study’s analyses also demonstrated that the combined effect of ICT investments and older workers led to an increase in productivity in Japan for high- and low-educated workers, but in Korea only for low-educated workers. The authors concluded that organizations can alleviate productivity decline due to aging by increasing ICT investments. This means that investing in ICT capital and technologies can potentially increase productivity and extend the working lives of older people.

In a rapidly changing world knowledge sharing is essential. One paper looked at generational differences and the use of social media for work purposes and found that beyond age, organizational rank, knowledge needs, enthusiasm, and personality played a role in influencing workers attitudes to use social media for knowledge sharing [31].

Min and colleagues [32] argued that Industry 4.0 has led to nonstandardized employment globally, such as gig work and short-term contracts, which makes OHS more difficult to implement and monitor. Overreliance and trust in new technology run the risk of large-scale and new types of accidents. The authors call for the development of new concepts of decent work, organizing of networks among independent workers to allow for OHS monitoring, and standardization of OHS regulations.

### A Multidimensional Perspective to Optimize an Age-Inclusive Workforce

Traditionally, aging is viewed by chronological age. However, organizations should think beyond the traditional concept that aging equals chronological age and instead employ a wider multidimensional perspective in the workplace. North [13] argues that generation, age, tenure, and experience must be integrated together to improve our understanding of aging workforce. The American Association of Retired Persons (AARP) uses the following lenses of aging at work: organizational tenure, career stage, life events, generation, accessibility, and chronological age (Table 1) [9]. The AARP did not include experience, like North [13] suggested, in their multidimensional perspective of aging. Examples of how this can be applied in practice are presented in Textbox 1.

**Table 1:** Multidimensional lenses of aging workforce.

Perspective	Explanation	Example
Organizational tenure^a,b^	Time spent within the same organization	2 years
Career stage^b^	Stages may include entry, establishment, advancement, maintenance, transition, and maturity (ISO^c^ 30400:2016) [33], or simply early, mid-, and late career.	Mid-career
Life events^b^	Age-related life events such as studying, getting married, having a child, becoming a carer for spouse or parents, being a grandparent or death of partner.	Carer for parents and children
Generation^a,b^	Specified birth cohort. Determines world views, experience of historical and economic events	Gen X
Accessibility^b^	Physiological or mental changes that impact the ability to work	Back problem and early onset dementia
Chronological age^a,b^	Years since birth	45 years
Experience^a^	Experience acquired in specific skills overtime	Years of online teaching

^a^Adopted from North et al [13].

^b^Adopted from American Association of Retired Persons (AARP) [9].

^c^ISO: International Organization for Standardization.

**Practical examples of different lenses of aging at work applied in practice.**

**Generation**

- Consider the different usage patterns of social media by different *generations* to ensure a wide pool of suitable applicants during recruitment [31].

**Organizational Tenure and Experience**

- An organization plans an expansion of an existing digital decision-support system. It is likely that those who have been longer with the organization or who have more *experience* with the system require less training, and hence, tailoring the training to specific needs may prove beneficial.

**Experience**

- During the initial outbreak of COVID-19, academic teaching staff with online teaching experience had less anxiety and required less training in transitioning their entire study units to online teaching than people who had less experience. The experience of seasoned online teachers was used to assist novices and was certainly not reflective of just age.

**Others**

- Other lenses that can be considered are biological, psychosocial, functional, and social aging [34]. Using different lenses when considering the aging workforce will ensure solutions are tailored to achieve optimum outcomes.

### How Can International Standardization Contribute to a Sustainable Aging Workforce?

#### What Is the International Organization for Standardization?

The ISO produces voluntary international standards. Its members are national standards bodies representing each country. ISO works closely with the International Electro Technical Commission (IEC). A standard is defined as a document that “provides requirements, specifications, guidelines or characteristics that can be used consistently to ensure that materials, products, processes and services are fit for their purpose.” [35].

The economic benefits of international standards are global harmonization of products and services, which increases trade, efficiency, and productivity. It also creates trust that products are safe and reliable among consumers, organizations, and governments. Each standard clearly identifies which Sustainable Development Goals it contributes to, thus reflecting their social and ethical benefits.

International experts contribute to working groups, which form part of technical committees. Working groups prepare the international standards. International standards go through several global commenting and voting stages to ensure consensus and practice-based solutions are reached. Experts are nominated by their national standards body and represent various stakeholder views including industry, small business, unions, academics, governments, consumers, or not-for-profit organizations.

The most relevant standard in relation to aging workforces will be presented below, while other standards will be listed where appropriate.

#### Technical Committee 314 on Aging Societies

Aging workforce was identified as a priority by the Technological Committee 314 on Aging Societies, resulting in the establishment of the first working group: *Working Group 1—Guidelines for an Age-Inclusive Workforce.* Approximately 16 countries from Europe, North America, Asia, Africa, and the Pacific participated. This international standard provides guidelines that allow organizations and other stakeholders to develop, implement, maintain, and support an age-inclusive workforce, while adding value to the organization, the older workers, communities, and other stakeholders [36]. Organizations can use this guideline as a stand-alone document or as part of their management systems, human resources activities, corporate social responsibility, or diversity and inclusion programs.

Digitalization of workplaces, digital literacy, and innovation form important elements. Recommendations in relation to digitalization and digital literacy focus, for example, on policies and procedures, remote working, training, resources, access, and mechanisms for evaluating technical opportunities.

Innovation recommendations encourage, for example, age-inclusive cocreation and co-design initiatives, where older workers are involved in workplace design, co-design, and create new products and services such as health apps. The rapidly aging demographic brings new, large, and untapped market opportunities. The added benefit of being creative at work is that evidence shows it also improves work engagement [37]. Tasks needing creativity, social intelligence, and human contact will remain and likely not be replaced by automation processes. Importantly, these tasks can be performed by people, regardless of their age [14].

The standard also addresses knowledge management and intergenerational collaboration. Both are relevant for an aging workforce to allow knowledge transfer between different generations and ICT skill building. Knowledge management systems not only include organizational knowledge management culture, structure, governance, and leadership but also roles and responsibilities, technology, processes, and operational matters [38]. ICT plays a relevant role in knowledge management systems.

#### Corporate Social Responsibility

Organizations that wish to operate in a socially responsible way and demonstrate commitment to sustainability can use the popular ISO 26000 standard guidance on social responsibility [39] as a guide to integrate social responsibility into their values and practices. Interestingly, there is a lack of organizations that have focused specifically on age from a diversity and inclusion strategic perspective. A 2015 PricewaterhouseCoopers CEO Global Survey found that 63.9% (549/858) of organizations had diversity and inclusion strategies, yet only 7.9% (68/858) had an age-inclusive strategy [40]. Organizations aiming to develop their aging workforce can capitalize on this by defining this goal as a corporate social responsibility activity, increasing their market competitiveness.

### How Can Ethics Contribute to a Sustainable Aging Workforce in the Area of Technology?

#### Overview

The rapid changes in technology and aging workforce require a continuous rethink in ethical principles and applications. For example, how do organizations act in a socially responsible way? How do we make AI systems trustworthy and how do we deal with their risks? How can organizations adapt their governance and consider these new risks?

#### Ethical Frameworks

Several ethical frameworks have been developed based on user group (eg, older users) or technology type such as AI, and these can help organizations manage their risks. Globally agreed standardized ethical principles are important to reduce trade barriers, allowing for exporting and importing of technology, and to build confidence in the ethical use of technology systems. Organizations will benefit from identifying an ethical framework relevant to their context and needs. Two examples are provided below, followed by the description of the trustworthiness of AI systems for an aging workforce.

First, an ethical framework for standardization of product and services in ICT and active and healthy aging consists of the following principles: accessibility and usability, affordability, autonomy and empowerment, beneficence and nonmaleficence, care protection and support, equality/equity and justice, inclusion/nondiscrimination and social impact, interoperability, and privacy/safety and security [41]. These ethical principles could easily be used to further guide aging workforce technological developments to reduce risks.

Second, leading technology companies and several international standardization bodies such as the ISO, the Institute of Electronic and Electronic Engineers (IEEE), and the Organization for Economic Cooperation and Development (OECD) [42] have recently developed AI ethical frameworks to deal with the risks surrounding AI. Of particular note is the G20 (a multilateral forum of the top 20 major economies in the world) adopted human-centered AI Principles in June 2019 [42], which were derived from the OECD AI Principles and include (1) inclusive growth, sustainable development, and well-being; (2) human-centered values and fairness; (3) transparency and explainability; (4) robustness, security, and safety; and (5) accountability.

#### Trustworthiness of AI Systems for an Aging Workforce

ISO has a Technical Committee (ISO/IEC Joint Technical Committee 1/Subcommittee 42 Artificial Intelligence) that produces AI-related standards. To make AI systems trustworthy and deal with their risk, organizations should adapt their governance and consider newly emerging risks. An example of such a new risk is unfair bias toward older workers based on age. This may take the form of agism in search engine algorithms or this may occur when unrepresentative training sets are used for machine learning systems [43] or AI-assisted decision making (eg, if only younger workers’ training records are used to predict performance). If there is an unfair bias, this is going to be exacerbated by AI systems. Therefore, organizations that use tools that consider bias when designing products and services may be able to rethink their approach concerning vulnerable populations. One such tool has been developed by the ISO and the IEC in 2020 as an international standard that can be used to guide organizations in ensuring their AI systems are trustworthy [44] and reduce bias: *ISO/IEC Technical Report 24028:2020: Information technology—Artificial intelligence—Overview of trustworthiness in artificial intelligence*. Trustworthiness can include, for example, reliability, availability, resilience, security, privacy, safety, accountability, transparency, integrity, authenticity, quality, and usability. The standard discusses approaches to establishing trust in AI systems “through transparency, explainability, controllability, etc.” Potential mitigation techniques and methods to combat engineering pitfalls and threats and risks to AI systems are also discussed in this standard.

Another available tool on the market is the P7000 standard, developed by the Institute of Electrical and Electronics Engineers (IEEC). This standard includes a process model that engineers and technologists can use to tackle ethical considerations when initiating, analyzing, and designing a system. The standard addresses the following *process* requirements: “management and engineering view of new IT product development, computer ethics and IT system design, value-sensitive design, and, stakeholder involvement in ethical IT system design.” [45].

Age bias in AI development in the workplace can further be avoided through inclusive practices. A system’s bias can emerge in perception, information processing, decisions, and through the design [43], and organizations should be aware of this when implementing ICT systems. For example, the design of robots mimicking characteristics of toddlers or young children (eg, big open eyes, voice). By using age-inclusive practices, the needs of aging workers can be included in the development and use of AI systems.

### How Can Blockchain and IoT Contribute to a Sustainable Aging Workforce in the Area of Technology?

#### Blockchain Data Management and the Aging Workforce

A 2019 systematic review has highlighted the different types of blockchain applications and identified human resources data management as an area of application [46]. More advanced forms of data protection in the form of blockchain are already tested [46]. Blockchain is an information recording system that has the potential to provide secure, pseudonymized, and immutable records of distributed information and is thought to potentially have a large positive social impact such as reducing age bias during recruitment processes or learning and development targets in an organization to ensure equal training opportunities across ages. To prevent misuse of the technology, blockchain should also be designed with ethical considerations in mind, and especially what impact it has on aging workforces and vulnerable and marginalized populations. While blockchain is able to restore personal control over data, it could also be misused to exert power over people and information. Therefore, blockchains should be intentionally designed by an ethical approach including reflections on governance, identity, verification and authentication, access rights, and ownership of data [47]. It is important to be age inclusive when developing such systems.

A 2019 systematic review reported that the use of blockchain has clearly enhanced data management and hence audibility because all operations can be verified [46]. However, full operability to allow cross-organizational management is still in its infancy but some prototyping of cross-organizational management of workflow is in development. Furthermore, there is still concern about its use due to data being stored on a public ledger and more research is needed to demonstrate real-world blockchain application [46]. We suggest that aging workforce and blockchain management form part of this research agenda. To this effect, Salah and co-workers [48] investigated blockchain applications in human resource management by interviewing human resources management experts. They identified potential applications may be useful for performance appraisal; recruitment; and verification of references, medical records, criminal records, or trainer credentials [48]. Other proposed blockchain-based credentialing are health care provider data management and directory systems [49]. The latter example attempts to match health care provider details with their national medical licenses. However, perceived blockchain adoption challenges are, for example, fear of layoffs, employee resistance due to lack of blockchain competence, worldwide adoption, support, and funding [48]. Overall, no application was found to improve the aging workforce and blockchain has yet to find its way into human resources management and thus aging workforces.

#### Integration of Blockchain With the Internet of Things and the Aging Workforce

Beyond blockchain, Mackey and colleagues [49] argue that in Japan’s case, integrating blockchain into the IoT ecosystem will be a future use case that will improve delivery of home care and telehealth services, particularly because of the growth of connected medical devices and smartphone apps. This may have 2 implications for our aging workforce. First, this integration will be essential to improve efficiencies and reduce cost for organizations that wish to support workers health and well-being and will also resolve health care workforce capacity problems [49]. Second, the integration will allow for improved self-management of chronic diseases, which has a direct impact on the aging workforce. For example, continuous glucose monitoring wearables for patients with diabetes or flash glucose sensor technology, which do not require a fingerpick to measure insulin levels, will allow people to manage their diabetes more efficiently, and thus increase their ability to continue to work. It is expected that wearable technology will continue to improve the aging workforce. Organizations could benefit from considering implementing or funding wearable technologies for their workers, for example, in the form of benefits as part of an employee contract.

### How Can Aging Workforce ICT Research Contribute to a Sustainable Aging Workforce?

#### Overview

The synergy of international standardization and ethical framework tools with research can advance the work in improving aging workforces. We present an example of an international research project below.

#### Ageing@Work Vision of AI-Enabled ICT Solutions

Ageing@Work is a European Union–funded international research project that aims to develop a set of adaptive and personalized ICT tools to help aging workers to maintain their work ability and enable them to be active and age healthily. An aging-appropriate work design will be created through the use of AI, augmented reality, virtual reality, and virtual assistant systems. The Ageing@Work toolkit will be developed through user-centered design and pilot tested in 2 sites in core Industry 4.0 processes [50]. The first pilot is a German indoor, machining factory. The workplaces are located in an industrial hall at different machines, which need to be equipped with tools, programmed, and observed. Normally, the worker must observe several machines simultaneously. The second pilot is a Spanish outdoor, mining factory. The workers of the quarries and treatment factory have to handle heavy machinery in outdoor conditions. They also carry out physically demanding maintenance work on the machinery.

**Figure 1:**
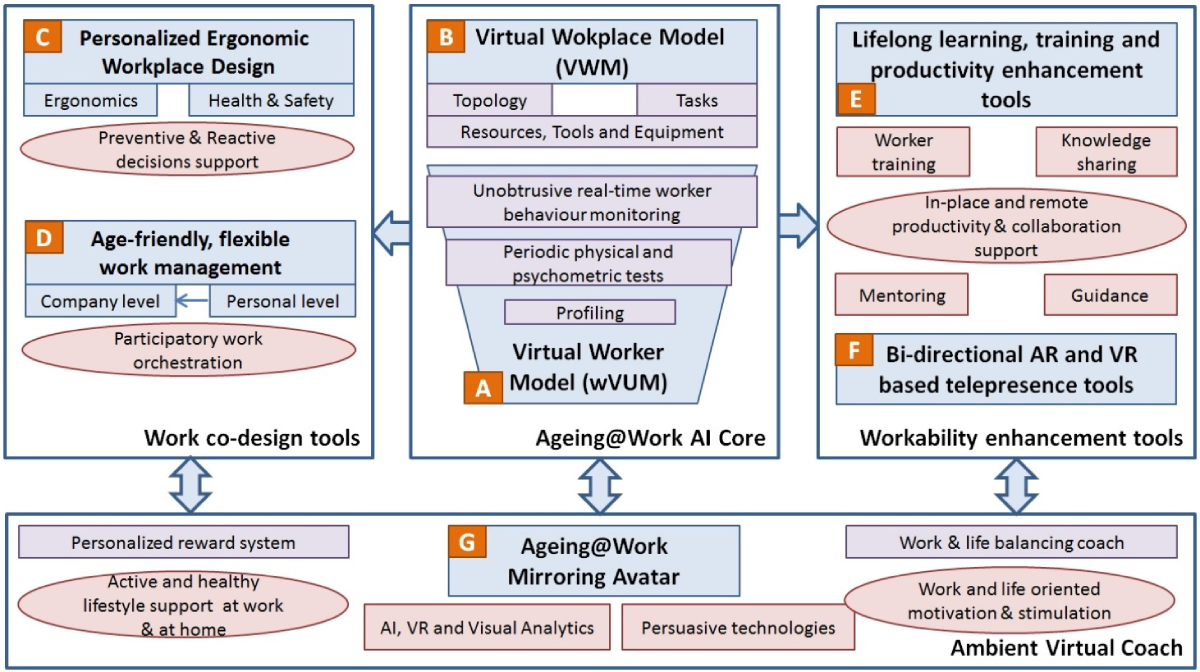
Ageing@Work concept [50]. AI: artificial intelligence; AR: augmented reality; PPE: personal protective equipment; VR: virtual reality.

The toolkit builds upon an AI core consisting of virtual user models and virtual workplace models that allow to adapt the work processes and the ergonomics of workplaces to the evolving needs of aging workers (see A/B in Figure 1). The virtual user models will include an activity monitoring system based on unobtrusive sensors such as smartphone apps or wearable sensors that will provide well-being data. As the system contains sensitive health data, it is subject to strict data protection rules. The virtual workplace models represent a virtual mapping of the work environment, that is, the arrangement of machines and equipment. Based on the data collected from aging workers and their workplaces, the system will be able to inform the workers about possible individual interventions. The system will also inform the organization about general improvement possibilities in work processes and task assignments as well as in ergonomics and health and safety of the work environment.

To adjust the workplace to the changes in the functional capabilities of aging workers, a personalized ergonomic design tool will be developed. This is intended to help minimize health risks by simulating improvement potential based on the virtual workplace models and enabling an ergonomic workplace design (see C in Figure 1). The work processes will be improved by a work decision-support tool that makes recommendations for the task assignment that are flexible and adjusted to the worker’s needs. Based on the collected data from the worker and the work processes, the system makes personal recommendations to individual workers for optimized task scheduling and informs managers about possible improvements to the entire process (see D in Figure 1). The system guarantees that managers will not receive personal data on individuals, but only an aggregated evaluation.

A range of productivity enhancement tools developed based on virtual and augmented reality will support the productivity and work ability of the aging workers. These include extended telepresence tools that enable older workers to collaborate remotely with younger workers and support virtual demonstration of work tasks. In addition, knowledge management tools for lifelong learning and knowledge sharing will be provided, which allow both older and younger workers to acquire knowledge for learning new tasks and to keep the experience of older workers in the organization and pass it on to younger workers (see E/F in Figure 1).

Furthermore, a virtual assistant tool, the Ambient Virtual Coach, will provide an easy-to-use interface consisting of an empathetic, mirroring avatar that makes recommendations on the work processes and the workers behavior based on the information from the virtual user and workplace models (see G in Figure 1). Combined with a personalized reward system, the Ambient Virtual Coach will motivate positive behavior at work and home by promoting work-life balance and quality of life.

The Ageing@Work project integrates novel ICT tools in core Industry 4.0 and is multidisciplinary, covering ergonomics, psychology, and behavioral research. It combines the design of aging-appropriate work systems with the role of motivation among aging workers in improving their positive behaviors and perceptions about work. Using a wide range of advanced technology in combination with a virtual avatar, Ageing@Work pursues the goal of a highly personalized support for active and healthy aging in the context of an improved workplace’s adaptation and productivity [50].

### Use Cases to Sustain an Aging Workforce

#### Overview

To assist organizations in creating good working conditions and identifying suitable technology to enable adaption to an aging workforce, several use cases will be discussed below. Some cases to sustain an aging workforce have been in use for several decades and have been continuously developed and adapted to changing conditions, such as the Exposure-Documentation-System, and some cases describe newer technologies, which have just recently found their way into organizations or are still in testing stage.

#### Exposure-Documentation-System

The multilingual “Belastungs-Dokumentations-System (Exposure-Documentation-System)” is an ergonomic assessment tool that allows organizations to systematically assess work-related exposures of aging workers, derive preventive measures, and subsequently design aging-appropriate work systems. It has been successfully implemented in operational practice for many years and supports companies in creating jobs, independent of age, that can be performed by aging workforces [51].

The Exposure-Documentation-System is based on the occupational science procedure “Beurteilung Arbeitsbedingter Belastungen (Assessment of work-related exposures),” which has been continuously refined since 1977. It has been adapted to validated ergonomic findings and current needs of organizations in many industrial sectors such as iron and steel, glass and ceramic, trade and goods logistics, forwarding agencies and port handling, and chemical and automotive industries as well as small- to medium-size enterprises. It supports companies in occupational health management, occupational integration management, the simulation of future work systems and the assessment and design of working conditions, demographic change, and the retaining of skilled workers in the company.

**Figure 2:**
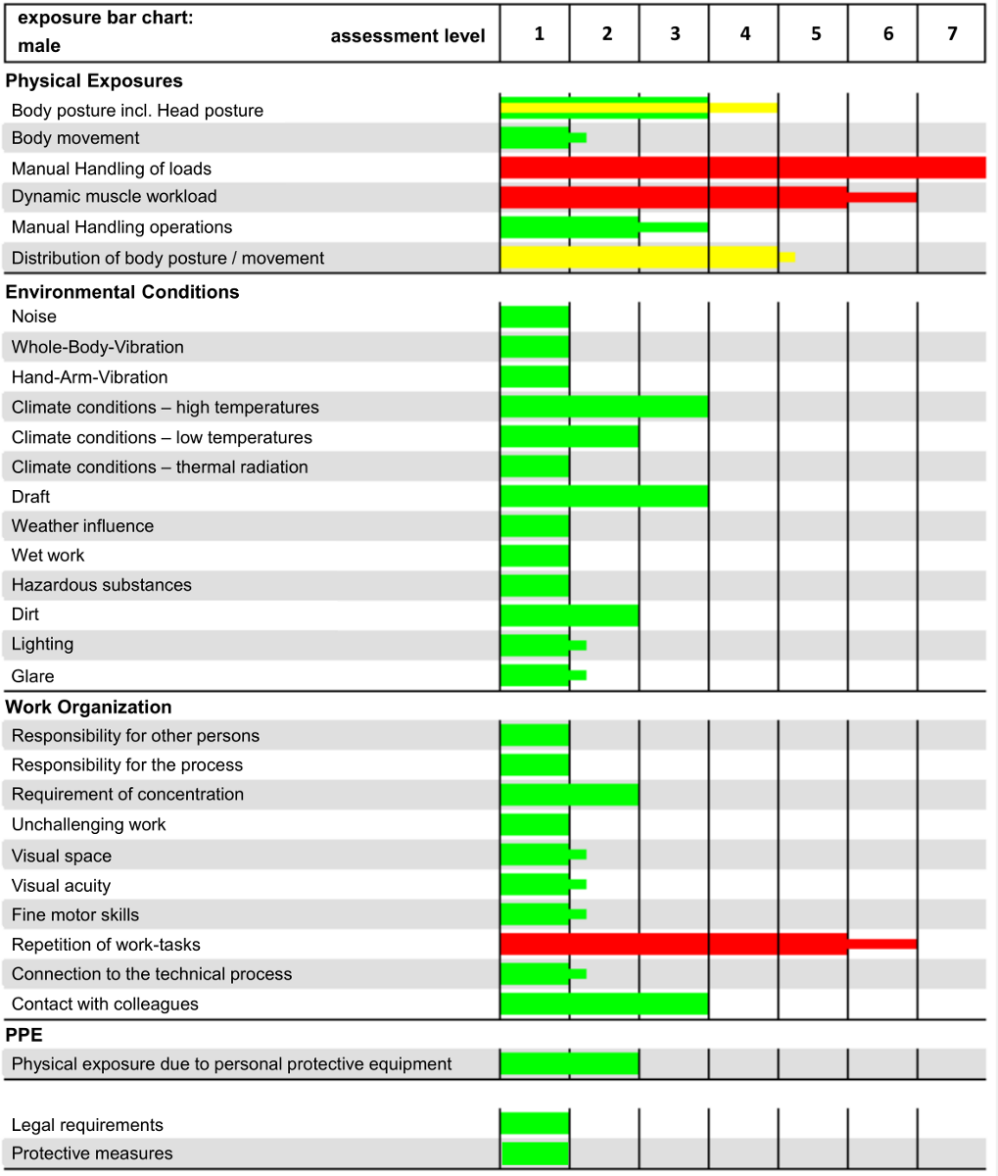
The Exposure-Documentation-System workplace profile with demographic analysis for aging workers. Incl: including; PPE: personal protective equipment.

Work-related exposures can be assessed on the basis of workplace observations and measurements to derive appropriate design measures. Physical and mental workloads, environmental conditions, and occupational safety are recorded by trained personnel and scientifically analyzed by the Exposure-Documentation-System. The workplace analysis is process oriented, that is, the data collection is based on individual work tasks and collected during a workers’ shift. The individual workload assessments are combined by the Exposure-Documentation-System to form a workplace profile that reflects the worker’s exposures over the entire shift (Figure 2).

The systematic analysis of physical and mental exposures can identify ergonomic improvements. The system enables a standardized exposure assessment to measure ergonomic quality with more than 30 items on a 7-point scale ranging from 1 (very low exposure) to 7 (overload very likely). The results are displayed in a traffic light system to illustrate the strengths and weaknesses of the analyzed work system. A green bar means that overall the exposures are harmless to the worker’s health. A yellow bar shows the maximum acceptable exposure value, that is, the workplace is only suitable for appropriately trained and healthy workers. A red bar means that the limit of acceptable exposure is exceeded and therefore requires action.

Design measures can be identified based on the assessment. The Exposure-Documentation-System also allows the tracking and verification of the effectiveness of the measures taken. This may help organizations to find appropriate technical tools for sustaining an aging workforce, for example, by determining the possible applications of collaborating robots in the workplace and their impact on the working conditions of aging workers.

Based on scientific findings, the Exposure-Documentation-System evaluates the higher bodily exposure of older workers and younger workers resulting from the same workload. The differences in the assessment are reflected in the workplace profile, with the thin bars representing the stress levels for older workers (Figure 2). Aging workers will have a higher total dose of exposure [52]. However, it is recommended that this be considered equally for younger workers.

Beyond the prescribed health and safety requirements in the workplace, the Exposure-Documentation-System enables organizations to create attractive jobs for aging workers. This supports them in increasing work motivation and satisfaction and has a positive effect on the organizational competitiveness.

#### Wearable Technology for Older Workers

Wearable technology is increasing in popularity. A gray literature review conducted by the Aged Care Industry Information Technology Council reported that wearable technology in the aged care sector is on the rise [53], which presents new opportunities for the aging workforce. A substantial proportion of the older population or people with a chronic condition are at an increased risk of COVID-19, and this has serious workforce implications such as people being required to work from home or at-risk people being directed to change roles or work in a different department. Wearable technology allows at-risk workers or those currently under quarantine or self-isolation to self-monitor for symptoms remotely, thereby protecting the health of the workers.

For example, the LifeSignals Biosensor 1AX [54] is a wireless medical biosensor and COVID-19 personal symptom monitoring device that when placed on the patient’s chest monitors symptoms and reports these (in real time) to the LifeSignals App. The biosensor is linked with a smartphone app and allows for continuous monitoring of COVID-19 symptoms. It is for single-use only and can be used for 5 days for the early detection and monitoring of COVID-19 symptoms [55]. The patch records 2-channel electrocardiogram, heart rate, respiration rate, skin temperature, and motion (via an accelerometer). The app shows and tracks the vital signs in real-time, using easy-to-follow traffic light charts, similar to the Exposure-Documentation-System. The app provides health trends and alerts so the person can contact a health care provider if required. The technology is interoperable and can be integrated into other platforms, systems, and apps. The app is also compliant with the international standard ISO 13485:2016 Medical devices—Quality management systems—Requirements for regulatory purposes [56].

It is expected that wearable technology will continue to improve the aging workforce, depending on the application. Despite the potential of wireless biosensors, the acceptance by an aging workforce remains to be seen and the concerns around ethics discussed above remain. However, organizations could consider offering wearables, such as a biosensor, for those who wish to use it. Organizations could benefit from considering implementing or funding wearable technologies for their workers, for example, in the form of benefits as part of an employee contract. Additionally, the workers can choose not to share the data from the app with their employer yet still improve their health.

#### Digital Voice Notes Across Applications for Aging Workers

Another opportunity for aging workers is the digital voice technology. It enables quick communication with co-workers via real-time voice chat and allows for leaving messages across different applications [57]. This is useful for faster communication, decision making, having fewer meetings, and better understanding the voice tone of the sender of the message, which improves mutual understanding. This technology is specifically beneficial for aging workers for several reasons. First, it is specifically useful for aging workers who may have trouble typing messages fast due to dexterity issues or who simply forgot their reading glasses. Instant voice chat can resolve this issue instantly. Second, verbal fluency remains at a high functional level until advanced ages [14], therefore the use of digital voice notes benefits especially aging workers. Third, for lower-educated older workers the transition to digital technology by using voice notes rather than requiring to type notes will potentially increase acceptance of such technology at a faster rate and thereby their employability to remain in the workforce as digitalization of workplaces continues.

### Recommendations

Based on the review of international standardization practices, collaborative research projects, and uses cases, we propose the following ICT-related recommendations (Textbox 2) that organizations could consider to get started in improving their aging workforce.

**Recommendations.**

**Perspectives**

- Apply a multidimensional perspective to optimize an age-inclusive workforce in your organization (eg, organizational tenure, career stage, life events, generation, accessibility, chronological age, and experience).
- Identify how the skills of younger and older workers complement each other rather than substitute each other.

**International Standards**

- Investigate which international standards are relevant to your organization or your project to improve an aging workforce from a human resources management or information technology perspective.

**Human Resources–Related International Standards**

- Be a leader in becoming an age-inclusive organization
  Become involved with the ISO (International Organization for Standardization) TC (Technical Committee) 314 Aging Societies community [58].
  Follow the International Standard ISO 25550:2022 Aging societies—General requirements and guidelines for an age-inclusive workforce [34].
- Promote sustainable employability among all staff.
  Consider using the following international standard as a guide: ISO/TR (Technical Report) 30406:2017 Human resource management—Sustainable employability management for organizations [59].
- Promote knowledge management and intergenerational collaboration to allow knowledge transfer between different generations in the aging workforce.
  Consider using the following international standard: ISO 30401:2018 Knowledge management systems—Requirements [38].
- Promote age diversity in the workplace.
  Operate in a socially responsible way and demonstrate commitment to sustainability through the ISO 26000 Standard guidance on social responsibility [39] or ISO 30415:2021 Human resource management—Diversity and inclusion [60] with a focus on being an age-inclusive organization.

**Technology-Related International Standards**

- Understand risk, such as age bias, and risk-mitigation practices of emerging technologies by consulting international standards. For example,
  ISO/IEC (International Electro Technical Commission) Technical Report 24028:2020 Information technology—Artificial intelligence—Overview of trustworthiness in artificial intelligence [44].
  P7000 Engineering methodologies for ethical life-cycle concerns [45].
  ISO 13485:2016 Medical devices—Quality management systems—Requirements for regulatory purposes [56].

**Ethics**

- Benefit from identifying an ethical framework relevant to organizations’ context and needs. Many frameworks are available as either general or specific guidelines based on user group (eg, older people) or technology used.
- Choose and implement new technologies carefully and identify ethical and legal issues and take appropriate technical and organizational measures in advance of data collection or processing.
- When designing technology to improve your aging workforce, apply universally agreed ethical principles based on aging principles or technology principles. For example,
  The ethical principles of standardization in information and communication technology (ICT) and active and healthy aging: Accessibility and usability, affordability, autonomy and empowerment, beneficence and nonmaleficence, care protection and support, equality/equity and justice, inclusion/nondiscrimination and social impact, interoperability, and privacy/safety and security [41].
  The G20-adopted human-centered artificial intelligence principles [42]: (1) inclusive growth, sustainable development, and well-being; (2) human-centered values and fairness; (3) transparency and explainability; (4) robustness, security, and safety; and (5) accountability.
- Avoid age bias in ICT development and implementation.
- Involve older people in the planning, development, and implementation of ICT applications.

**Research**

- Consider using new technologies to create an aging-appropriate work such as artificial intelligence, augmented and virtual reality, and virtual assistant systems.
- Ensure technologies are developed through user-oriented design, and consider the needs of aging workers and the changes in functional capabilities before implementing new technologies.
- Promote work ability of aging workers by adapting the work processes and the ergonomics of workplaces to the evolving needs of the aging workers and encouraging healthy behavior.
- Enable older workers to collaborate remotely with younger workers so that they can work from home.
- Provide knowledge management tools for lifelong learning and knowledge sharing, which allow both older and younger workers to acquire knowledge for learning new tasks and to keep the experience of older workers in the organization and pass it on to younger workers.
- Multidisciplinary research is needed to identify opportunities and challenges for blockchain use and other technologies in the area of aging workforce.

**Use Cases**

- Systematically assess work-related exposures of aging workers and derive preventive measures to design aging-appropriate work systems.
- Consider higher-workload exposures of older workers equally for younger workers, due to the higher total dose of exposure.
- Track and verify the effectiveness of measures taken (before implementing new technologies) to identify whether there has actually been an improvement for aging workforces.
- Consider promoting, implementing, or funding wearable technologies for aging workers, for example, in the form of benefits as part of an employee contract.
- Consider promoting or implementing digital voice notes across applications for aging workers.

## Discussion

### Principal Findings

This paper outlined the implications of aging on working populations in the context of Industry 4.0. Technology has known downsides such as job losses but can also have benefits such as keeping people at work through assisting older workers in undertaking physically demanding tasks. Understanding aging through different lenses, beyond simply age, is important to make real changes in an organizational and digital context. ISO has various existing or emerging standards that tackle the area of aging workforce and ICT applications, which are based on best practice and international consensus. These standards can guide organizations in identifying a way forward to manage their workforce [61] and the challenges brought on by ICT. These standards can also inform training institutions in curriculum development and competency training relevant to aging workforces, which, to our knowledge, is not commonly used currently, such as human resources management, ergonomics, and health informatics. Standards can also be used to train new types of workforces such as the rise in the telehealth workforce [7]. Organizations and governments should consider using the newly introduced international standards that focus on ICT and aging workers so they can improve and prolong working life of people, which may also lead to reduced organizational costs and an increase in retirement age. The following question remains though: “How ready the aging workforce is in embracing technology?” To answer this question, in 2020, the Aged Care Industry Information Technology Council Australia [62] conducted a national benchmark analysis of the technology readiness of the aged and community care sector. The authors found no good specific examples of ICT implementation to assist the older workforce; instead their research found a real need for better digital inclusion and digital workforce improvements across the board. The authors concluded that at a sector level, there is a need to address the significant variability in technology capabilities in terms of infrastructure as well as workforce expertise, and attitudes to technology-enabled care.

Furthermore, AI, robotics, digital voice notes, blockchain, IoT, and wearable technology can serve our aging workforces as has been described above. But when designing or implementing technology to improve aging workforces, organizations should always strive to improve the ergonomics of work systems and apply universally agreed ethical principles to manage associated risks. Additionally, organizations can leverage their identity as being an age-inclusive workforce through linking this to corporate social responsibility and be an employer of choice.

Building relationships with key stakeholders across disciplines, industries, government, industry, and research will also lead to increased identification of evidence-based solutions such as the Ageing@Work project described above or the MAIA project (Models and Methods for an Active Aging workforce: an International Academy), which has started in 2020 [63]. MAIA is a research and innovation staff exchange funded by the European Union’s Horizon 2020. The academy concentrates on the problems and needs of the aging industrial workforce. The academy is multidisciplinary including aging, psychosocial, ergonomics, manufacturing system design, robotics, assistive technologies, and economics. These projects can form the blueprint for other international collaborations to find common solutions to common problems.

Finally, we have provided a set of recommendations on how international standardization can be used to improve aging workforce productivity, health, and competitiveness through the use of ICT when designing work for aging populations.

It is recommended that age-inclusive practices, international standards, and research are combined to further improve the aging workforce. For example, a recent study measured emotional exhaustion among regional doctors in training and the application of international standard guidelines on sustainable employability management in a hospital. All criteria from the ISO sustainable employability guidelines that were measured were significantly associated with emotional exhaustion, demonstrating the applicability of the guideline. There is potential to incorporate standards that relate to aging workforce into future aging research to further improve the credibility and application of standards in practice. Given the rise of nonstandardized employment globally, such as gig work and short-term contracts, governments play a crucial role in ensuring the OHS of workers, through developing new concepts of decent work, organizing of networks among independent workers, and standardization of OHS regulations [32]. A recent study [8] reported case studies from 15 countries to address the impact of COVID-19 on aging workers and included many technological solutions such as next-generation age-inclusive manufacturing systems in Germany or an AI system run by a large Korean telecommunications company (SK Telecom) that continuously tracks the health of service users to reduce workload for health care staff.

In the wake of inadequate pension systems, reduced savings and low interest rates, a global financial crisis, pandemic, and increased divorce rates, many older people do not have sufficient income to retire and may be required to continue to work to survive. Currently, there is a lack of good road maps on how organizations can capitalize on their aging workforce and deal with the risks. Current business and employment models, practices, and policies are slowly changing to adopt to the new way of working. ICT has proven it plays a pivotal role in this area. Governments will play a role in assisting the adaption and implementation of technology, ethical frameworks, and international standards that support the aging workforce.

### Conclusions

Applying a multidimensional lens on aging in organizations such as organizational tenure, career stage, life events, generation, accessibility, chronological age, and experience will improve sustainable employability among aging workers. The synergy of international standardization and ethical framework tools with research and use cases can advance the work in improving aging workforces. Technological developments can support achieving an age-inclusive workforce, such as AI, virtual assistants, wearable technology, or blockchain solutions coupled with IoT. These developments will undoubtedly find a stronger place within the global context and is most likely to have increased acceptance of ICT applications among aging workers as well as organizations and governments. International standardization, using ethical frameworks and standards, cross-country research, and learning from use cases play an important role to ensure practical, efficient, and ethical implementation of ICT solutions to contribute to a sustainable aging workforce.

## Abbreviations

AARP: American Association of Retired Persons
AI: artificial intelligence
ICT: information and communication technology
IEC: International Electro Technical Commission
IEEC: Institute of Electrical and Electronics Engineers
IEEE: Institute of Electronic and Electronic Engineers
IoT: internet of things
ISO: International Organization for Standardization
MAIA: Models and Methods for an Active Aging workforce: an International Academy
OADR: old-age dependency ratio
OECD: Organization for Economic Cooperation and Development
OHS: occupational health and safety
